# Aggressive lactating adenoma mimicking breast carcinoma: A case report

**DOI:** 10.1016/j.ijscr.2020.03.047

**Published:** 2020-04-22

**Authors:** Huyen Thi Phung, Long Thanh Nguyen, Hung Van Nguyen, Chu Van Nguyen, Hoa Thi Nguyen

**Affiliations:** a Department of Medical Oncology 6, Vietnam National Cancer Hospital, Hanoi, Viet Nam; b Department of Oncology, Vietnam University of Traditional Medicine, Hanoi, Viet Nam; c Department of Oncology, Hanoi Medical University, Hanoi, Viet Nam; d Department of Oncology and Palliative Care, Hanoi Medical University Hospital, Hanoi, Viet Nam; e Quan Su Pathology Department, Vietnam National Cancer Hospital, Hanoi, Viet Nam

**Keywords:** Lactating adenoma, Breast adenoma, Breast cancer

## Abstract

**Introduction:**

Lactating adenoma is a rare breast tumor which is commonly found in late pregnancy or lactation period. Despite its benign nature, lactating adenoma might develop aggressively and can be misdiagnosed as breast cancer.

**Case presentation:**

We report a case of a 25-year-old female presenting with two large, ulcerative and bleeding breast masses. An open biopsy demonstrating lactating adenoma was considered discordant and surgery was performed to confirm the diagnosis.

**Conclusion:**

Careful clinical and histological evaluations are necessary to diagnose and manage lactating adenoma.

## Highlights

• Lactating adenoma is a benign “tumor of pregnancy” which typically occur in the third trimester or lactation period.
• In some cases, the clinical presentation of lactating adenoma might mimic breast cancer.
• Surgery to remove the tumor for careful pathological assessment might be required to confirm the diagnosis.

## 1 Introduction

Pregnancy and lactation are associated with many breast lesions due to hormone-induced physiological and pathological changes. Among such lesions, lactating adenoma is a rare benign condition which is commonly seen in young primiparous women during their second or third decades of life [1]. However, in some cases, it can grow rapidly to a large size and the symptoms of infarction may mimic malignant tumors and lead to diagnostic difficulty [2,3]. Notably, the coexistence of lactating adenoma and breast carcinoma has been reported in some instances [4,5]. Medical history, careful clinical examination and histological assessment by core biopsy or even surgery help to confirm the diagnosis. Here we presented a case with large and ulcerated breast lactating adenoma in a pregnant woman that had been clinically misdiagnosed as breast cancer. This work has been reported in line with SCARE criteria [6].

## 2 Case presentation

**Fig. 1:**
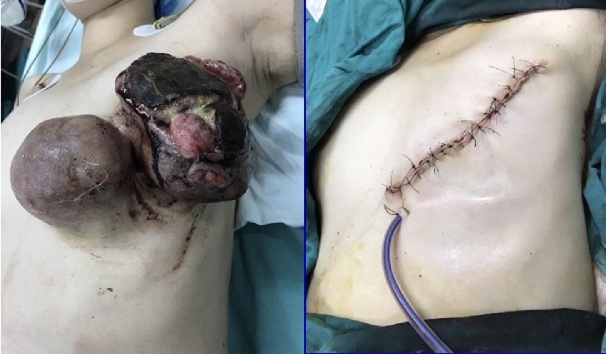
The patient presented with two large, ulcerative and bleeding breast masses. The tumors were then completely removed.

A 25-year-old female came to our institution after noticing a rapid growing mass in her left breast for 16 weeks. She had no past medical histories and was at the week 25 of her first pregnancy. On examination, the patient was afebrile and there were two large masses on her left breast with diameters of 20 × 7 cm and 10 × 6 cm, which was ulcerated and bleeding (Fig. 1). A relatively fixed 2-cm lymph node in the left axilla was also noticed. On ultrasound, there were two heterogeneously echogenic lesions with irregular borders and fatty infiltrate, of which the diameters were about 10 cm and 20 cm respectively. Besides, several axillary lymph node with the largest diameter of 2 cm were found, but the fatty hilum was still present. The patient was anemic, with a hemoglobin level of 58 g/L.

Local dressing and pressure were applied to slow down the bleeding. Blood transfusion and prophylactic antibiotics were also indicated. The initial clinical diagnosis was cT4bN2M0 breast cancer. However, an open biopsy with local anesthesia was done which yielded the result of lactating adenoma of the breast. The tumor still significantly bled and palliative mastectomy was performed. During surgery, we found two large tumors which had unclear borders and spread to almost entire breast tissue but did not invade the pectoralis muscles.

**Fig. 2:**
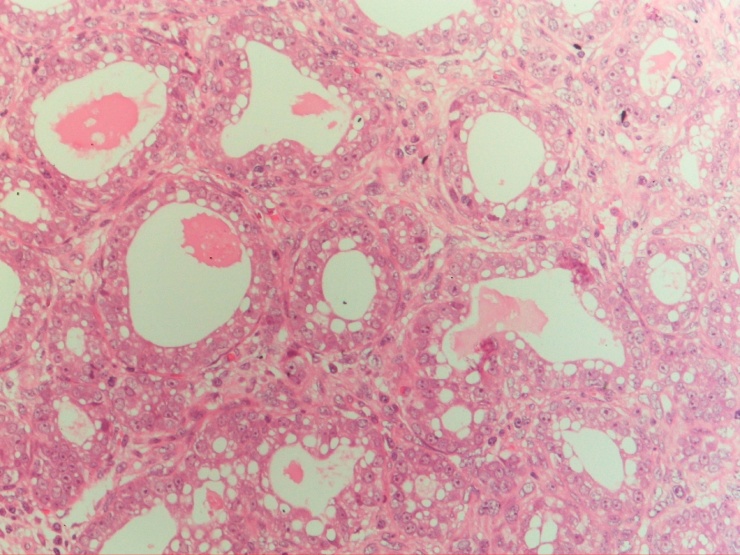
Microscopical picture showed proliferation of benign ducts lined with secretory epithelial cells with large vascuolated cytoplasm (HE × 200).

On post-operative pathological examination, there were necrosis areas along with breast ductal hyperplasia. The epithelial cells exhibited secretory changes which had mildly hyperchromatic, round nuclei and prominent cytoplasmic vacuoles (Fig. 2). The final diagnosis was then lactating adenoma. There were no post-operative complications, the axillary lymph nodes shrank gradually after antibiotic treatment and the patient was stably discharged two weeks later. The pregnancy then went well and she gave birth to a healthy girl after two months. After two years of follow-up, there have been no signs and symptoms of recurrence or metastasis.

## 3 Discussion

Lactating adenoma is a benign epithelial lesion of the breast which typically occurs in the late pregnancy and through the period of lactation. Some pathologists suggest that lactating adenoma is a distinct condition, while others consider it as a result of pregnancy-induced changes from pre-existing fibroadenoma, lobular hyperplasia or tubular adenoma [7]. Slavin et al. and James et al. propose that lactating adenomas are pure and separate breast lesions that are always related to current or recent pregnancy and are nodules of physiologic lobular proliferation, making it more prominent than surrounding normal tissue and forming a clinically detectable mass [8,9]. Therefore, lactating adenomas are histologically characterized by proliferation of benign ducts lined with typical secretory cells that contain large cytoplasmic vacuoles and lobular architectures are preserved. It can be distinguished from lactational changes of pre-existing fibroadenoma as such changes tend to be more localized and the features of fibrous component in the rest of the tumor are still present [1]. De novo origin of lactating adenoma is also supported by the findings that the immunohistochemical phenotype of lactating adenoma is similar to normal breast tissue of pregnant women, but different from that of tubular adenoma [10]. However, other authors suggest that tubular adenoma and lactating adenomas are variants of the same lesions, in which the latter arise from pre-existing adenomas under the physiological states of pregnancy [11].

Clinically, lactating adenomas usually presents as a firm, painless and mobile breast mass about 2–4 cm in diameter and is well-defined from the surrounding breast tissue [1]. The differential diagnoses might include phyllode tumors, focal mastitis or benign breast adenomas [2]. However, the tumor might grow rapidly to a huge size such as 27 × 18 cm as Elzahaby reported [12]. Hemorrhage and infarction can occur in approximately 5% of patients because of relative vascular insufficency [1]. In such cases, it can be misdiagnosed clinically as malignant breast tumor.

In our patient, the tumor also reached a very large size of 20 cm and infarcted with an ulcerative appearance, which was highly indicative of breast carcinoma, especially when axially lymph nodes were also detected. Although the open biopsy demonstrated lactating adenoma, the diagnosis of malignancy was still be suspected. However, there were significant bleeding and the pregnancy state did not allow a long surgery with general anesthesia. In addition, the axillary lymph node was quite large and relatively fixed on clinical examination, which might cause difficulty in performing complete lymph node dissection. Therefore, palliative mastectomy was done, without frozen section assessment. In case the post-operative pathological diagnosis was malignancy, neo-adjuvant systemic therapy could be used to facilitate a second-stage axillary lymph node dissection and sentinel lymph node biopsy could be tried.

Baker et al. also reported a lactating adenoma case with very aggressive presentation and surgical excision was performed for the definitive diagnosis despite previous benign core biopsy results [1]. In these case, biopsies were considered discordant and surgical removal was required to confirm the diagnosis, especially when the rare co-existence of lactating adenoma and carcinoma has been described in some case reports [4,5]. Similarly, according to Tuveri et al., nipple adenoma of the breast can present as an erosive nodule and clinically mimic Paget’s disease, as well as can even be misinterpreted histologically as a ductal carcinoma [13]. The rare concurrence of nipple adenoma and carcinoma has also been reported [13]. Therefore, being aware of these presentations and careful clinical and histological evaluations are necessary to diagnose such lesions.

In terms of treatment, since lactating adenoma has a low risk of recurrence, enucleation is the recommended therapy, especially in patients with ulceration and bleeding. Bromocriptine, a dopamine agonist to suppress lactation can be used preoperatively to reduce the size of the tumor [14].

## Declaration of Competing Interest

None.

## Funding

None.

## Ethical approval

The study was approved by our research committee in National Cancer Hospital, Hanoi, Vietnam.

## Consent

The publication of this study has been consented by the relevant patient.

## Author contribution

Huyen T. Phung: main doctor who treated the patient, revised manuscript.

Long T. Nguyen: took part in the operation, wrote manuscript.

Hung V. Nguyen: took part in the operation, wrote manuscript.

Chu V. Nguyen: pathologist who assessed the biopsy and surgical specimen, revised manuscript.

Hoa T. Nguyen: followed up the patient, revised manuscript.

## Registration of research studies

This is not a first-in-human study, thus it is not needed.

## Guarantor

Huyen T. Phung, M.D., PhD.

## Provenance and peer review

Not commissioned, externally peer-reviewed.
